# Muscle cramp susceptibility is associated with muscle‐specific modulation of human motoneuron discharge patterns

**DOI:** 10.1113/EP093881

**Published:** 2026-08-10

**Authors:** Timothée Popesco, Romain Dos Santos, Ilan Cassoli, Gabriel S. Trajano, Gregory E. P. Pearcey, Nicola A. Maffiuletti, Chris Donnelly, Bengt Kayser, Nicolas Place

**Affiliations:** ^1^ Institute of Sport Sciences, Faculty of Biology and Medicine University of Lausanne Lausanne Switzerland; ^2^ School of Exercise and Nutrition Sciences, Faculty of Health Queensland University of Technology (QUT) Brisbane Queensland Australia; ^3^ School of Human Kinetics and Recreation Memorial University of Newfoundland St. John's Newfoundland and Labrador Canada; ^4^ Human Performance Lab Schulthess Klinik Zurich Switzerland

**Keywords:** discharge rate hysteresis, high‐density electromyography, motor unit behaviour, neuromodulation, plantar flexors, spinal excitability

## Abstract

Muscle cramps are painful, involuntary contractions thought to arise from altered spinal motoneuron excitability, potentially involving persistent inward currents (PICs). We examined whether cramp susceptibility is associated with altered PIC‐related motoneuron behaviour and whether electrically induced cramp modifies these estimates. Twenty‐eight healthy, active adults performed triangular plantar‐flexor contractions before and after a cramp threshold frequency test designed to evoke cramp in gastrocnemius medialis. Participants were classified as crampers or controls according to cramp test outcome. Contributions of PICs to motoneuron discharge were estimated in soleus and gastrocnemius medialis from high‐density surface EMG recordings during triangular contractions decomposed into motor unit spike trains. Discharge rate hysteresis (Δ*F*), brace height, associated slopes and self‐sustained discharge duration characterized PIC‐related modulation of motor unit firing. Spinal reflex excitability and centrally mediated neuromuscular responses were assessed using H reflexes and wide‐pulse, high‐frequency neuromuscular electrical stimulation. At baseline, Δ*F* in gastrocnemius medialis was lower in crampers than in controls, whereas other PIC‐related estimates, wide‐pulse, high‐frequency‐evoked torque, sustained EMG activity, warm‐up effect and H‐reflex amplitudes did not differ between groups. After the cramp‐inducing protocol, Δ*F* increased in gastrocnemius medialis and self‐sustained discharge duration increased in both muscles, while brace height remained unchanged in gastrocnemius medialis but decreased in soleus. Maximal discharge rate in gastrocnemius medialis decreased in crampers but not in controls. These findings indicate that cramp susceptibility is not associated with generalized motoneuronal hyperexcitability, but with subtle, muscle‐specific alterations in motoneuron discharge modulation.

## Abstract (highlights)

- **What is the central question of this study?**
  Muscle cramps are thought to involve altered motoneuron excitability, potentially through modulation of persistent inward currents (PICs), but whether cramp‐susceptible individuals exhibit altered baseline discharge behaviour and distinct responses to a cramp‐inducing protocol remains unclear.
- **What is the main finding and its importance?**
  Cramp susceptibility was not associated with generalized motoneuron hyperexcitability, given that most PIC‐related estimates, centrally mediated responses to wide‐pulse high‐frequency neuromuscular electrical stimulation and spinal reflex measures were comparable across groups. Crampers showed altered gastrocnemius medialis, but not soleus, discharge modulation and reduced post‐cramp changes, suggesting localized alterations in motoneuron discharge behaviour rather than a global increase in spinal excitability.

## 1 INTRODUCTION

Muscle cramps are sudden, painful and involuntary contractions most frequently affecting the triceps surae muscle group. They are prevalent in healthy individuals, with epidemiological studies reporting that ≤60% of adults experience cramps during their lifetime (Katzberg, 2015), and remain a significant clinical concern owing to their unclear pathophysiology (Miller & Layzer, 2005; Schwellnus, 2007). Recurrent cramps have an important clinical and socioeconomic impact, contributing to decreased quality of life, functional impairment and increased healthcare consultations, particularly in the elderly population (Katzberg, 2015; Miller & Layzer, 2005); their prevalence and severity are increased in chronic conditions such as amyotrophic lateral sclerosis, diabetes or liver cirrhosis, where they are associated with sleep disruption and mobility limitation (Katzberg, 2015; Minetto et al., 2013).

Cramps are typically characterized by high‐frequency motor unit discharges, referred to as ‘cramp discharges’, that persist beyond voluntary or stimulus‐driven contraction (Harmsen et al., 2021; Minetto et al., 2013). Although cramps were historically attributed to dehydration or electrolyte imbalance (Talbott & Michelsen, 1933), growing evidence indicates that enhanced spinal motoneuron excitability and reduced inhibitory control are key players (Minetto et al., 2011; Schwellnus, 2009). In healthy individuals, cramps can occur in different contexts, including during or after exercise and at night (Katzberg, 2015). To investigate the mechanisms underlying muscle cramps in the laboratory, electrically induced muscle cramps have been used in previous studies (Behringer et al., 2014; Miller & Knight, 2009). The cramp threshold frequency (the lowest stimulation frequency that induces a cramp) is lower in individuals prone to cramps and is correlated with self‐reported cramp occurrence, supporting its use as an experimental marker of cramp susceptibility and possibly increased intrinsic motoneuron excitability (Behringer et al., 2018; Miller & Knight, 2009). Interestingly, spinal reflex excitability has been assessed through Hoffmann reflex (H reflex) modulation in response to both voluntary and electrically induced muscle cramps, with inconsistent results (Harmsen et al., 2021). The sustained motor unit activity observed after stimulation offset and cramp induction suggests the implication of persistent inward currents (PICs) in cramp generation and prolongation (Harmsen et al., 2021; Minetto et al., 2011). Motoneuronal PICs are intrinsic depolarizing currents mediated by voltage‐dependent sodium and calcium channels that amplify and prolong synaptic input causing self‐sustained discharge (Heckman et al., 2008). Recently, Beauchamp et al. (2025) demonstrated that PIC‐related prolongation of motor unit discharge is most pronounced at shorter muscle lengths, where muscle cramps usually occur and motoneuron excitability is enhanced, and that impaired motor unit derecruitment in such conditions might lead to involuntary self‐sustained contractions.

Although it is not possible to measure PICs directly in humans, several methods have been developed to estimate contributions of PICs to motoneuron discharge. For instance, wide‐pulse, high‐frequency neuromuscular electrical stimulation (WPHF) has been used as a non‐invasive tool to assess neuromuscular function. This technique consists of long pulse durations (∼1 ms) delivered at a high frequency (typically 100 Hz) to promote reflexive recruitment of spinal motoneurons through large‐diameter afferents (Collins, 2007). When delivered at low intensities [∼10% maximal voluntary contraction (MVC) torque], it can elicit a progressive increase in torque (‘extra torque’) and sustained EMG activity after stimulation, both of which are considered to be centrally mediated responses reflecting enhanced motoneuronal excitability, presumably through increased contributions of PICs (Donnelly et al., 2021; Popesco et al., 2024; Wegrzyk et al., 2015). Additionally, the ‘warm‐up effect’ observed when tendon vibration is applied concurrently with neuromuscular electrical stimulation has been proposed to reflect a progressive facilitation of motoneuron responsiveness, probably mediated by the facilitation of PICs (Mesquita et al., 2021). To quantify PIC‐related behaviour further, the decomposition of high‐density surface EMG (HD‐EMG) signals into motor unit spike trains allows analysis of motor unit discharge patterns, including paired motor unit analysis to characterize discharge rate hysteresis (∆F; Gorassini et al., 2002) and recently developed metrics, such as brace height, acceleration and attenuation slopes, which characterize the modulation and shape of motoneuron discharge rates with increasing effort (Beauchamp et al., 2023).

Despite preliminary evidence suggesting that PICs contribute to cramps, no study to date has examined whether individuals prone to muscle cramps (crampers) differ in their basal motoneuron excitability in comparison to non‐crampers. Moreover, it remains unclear whether an evoked cramp induces short‐term alterations in motor unit discharge behaviour and PIC‐related contributions to motoneuron discharge, which would indicate activity‐dependent modulation of motoneuron excitability and could help to distinguish predisposing mechanisms from cramp‐induced adaptations.

To address these questions, we recruited individuals prone or not prone to muscle cramps and assessed several complementary indices of motoneuron excitability and PIC‐related discharge behaviour before and after an electrically induced cramp. Thus, the aim of the present study was to determine whether cramp susceptibility is associated with altered motoneuron excitability at baseline and whether an electrically induced muscle cramp modulates the contributions of PIC estimates to motoneuron discharge behaviour differently between crampers and non‐crampers. We hypothesised that crampers would exhibit greater baseline PIC‐related motoneuron behaviour than non‐crampers and greater centrally mediated responses to WPHF stimulation. We also hypothesised that the cramp‐inducing protocol would increase PIC‐related indices, with larger post‐protocol changes in crampers than controls. Because prior studies report inconsistent H‐reflex modulation in relationship to cramping, H‐reflex outcomes were assessed exploratorily.

## 2 MATERIALS AND METHODS

### 2.1 Ethical approval

All participants gave written informed consent before participation. The study was approved by the local ethics committee, the Commission cantonale d’éthique de la recherche sur l’être humain du canton de Vaud (CER‐VD, protocol 2022‐01817), and conformed to the standards set by the latest revision of the *Declaration of Helsinki*, except for registration in a database.

### 2.2 Participants

Twenty‐eight healthy active individuals, including 14 with no history of cramps (4 men and 10 women; 25 ± 9 years of age; 66 ± 10 kg; 173 ± 8 cm) and 14 individuals with self‐reported cramp susceptibility (4 men and 10 women; 25 ± 5 years of age; 71 ± 15 kg; 172 ± 9 cm) participated. All participants were considered active, because they orally confirmed engaging in physical activity at least three times per week. Individuals were recruited as potentially prone to cramps based on reporting at least one cramp within the past 3 months, following prior studies using a 3 month recall window for cramp prevalence assessment (Blyton et al., 2012; Maisonneuve et al., 2016).

### 2.3 Experimental design

The study followed a cross‐sectional design, with one familiarization and one experimental session, separated by 2–7 days. All testing sessions were conducted on the dominant leg, identified as the preferred leg to kick a ball. Participants refrained from caffeine for 2 h and from exercising for 24 h prior to testing. Participants were classified as either crampers or controls using a cramp‐induction test during the experimental session, with individuals in whom no cramp could be elicited considered as controls.

### 2.4 Familiarization

Participants were familiarized with neuromuscular electrical stimulation, whereby single stimulation on the tibial nerve and 20 s WPHF trains on the triceps surae muscle belly were delivered at various intensities. Participants were accustomed to voluntary plantar flexions, including three or four trials of isometric MVCs and three or four trials of triangular‐shaped contractions with visual feedback.

### 2.5 Experimental session

The experimental protocol is shown in Figure 1a. Tibial nerve stimulation was used at rest to determine maximal M‐wave (M_max_) and H‐reflex (H_max_) amplitudes. H‐reflex recruitment was initiated at 10% of H‐reflex threshold and increased in 2–4 mA steps to determine H_max_ and the slope of the recruitment curve. After a series of submaximal contractions (five to eight repetitions at 50%–75% estimated MVC torque) to warm up, participants were instructed to contract the plantar flexors as hard as possible and to maintain the MVC for 3–5 s. Strong verbal encouragement and real‐time visual feedback (torque trace) were provided to maximize effort and ensure reproducibility. Superimposed and potentiated twitch responses were delivered respectively during and ∼2 s after the second and (if needed) third MVC for calculation of voluntary activation. Criteria for validity included: plateau at peak torque, no further torque increase after superimposed twitch, and <5% difference between the two last MVC trials.

**FIGURE 1:**
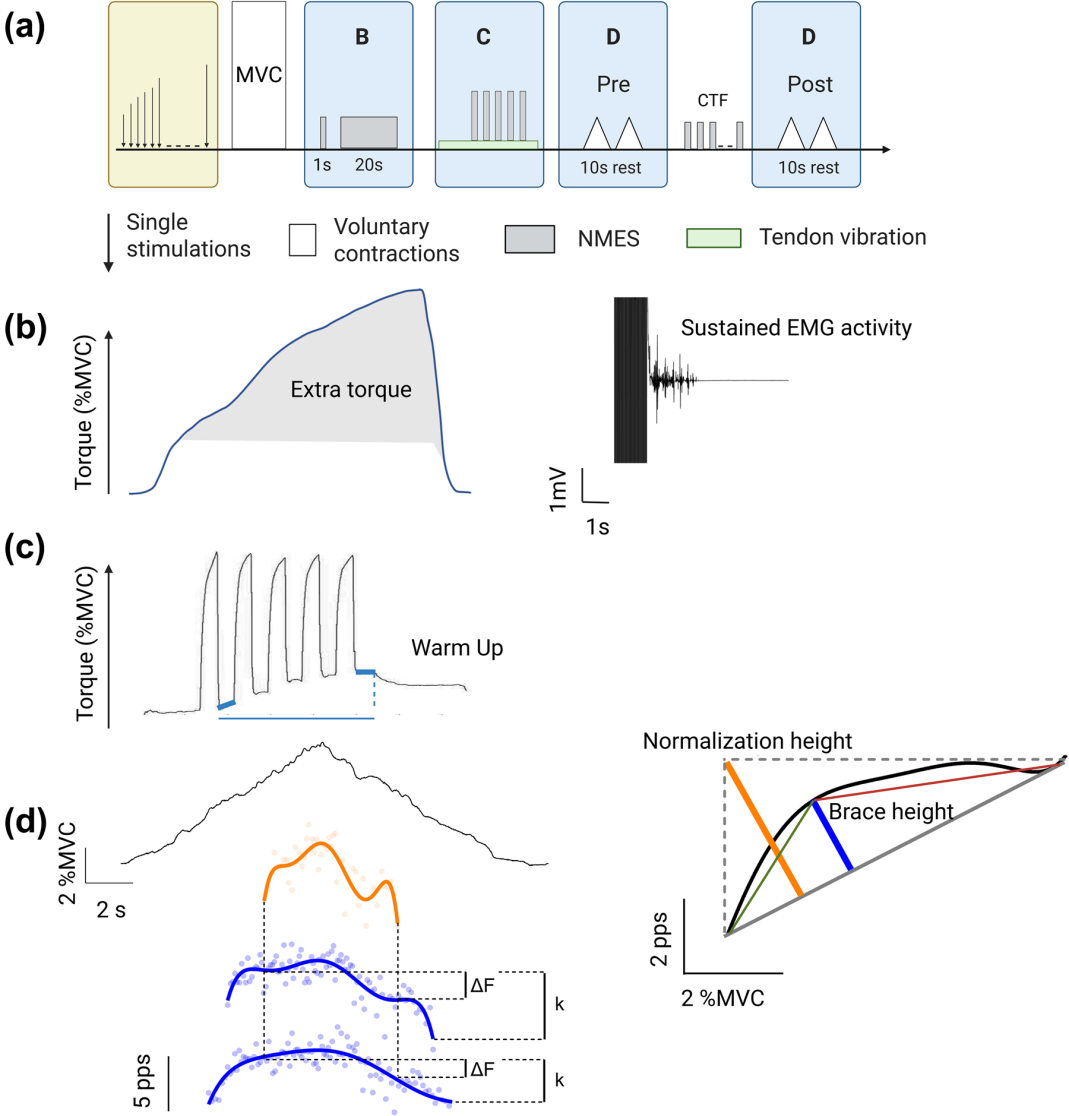
Study outline. (a) Single stimulations were used to evoke H reflexes and M‐waves in soleus and gastrocnemius medialis muscles. Boxes B, C and D show estimates of PICs. (b) Extra torque and sustained activity measurements during and after a 20 s WPHF train. (c) Representation of the warm‐up effect, quantified as the difference in torque between the two blue lines (*T*_1_, left and *T*_vib_, right). (d) Visual representation of the paired motor unit analysis (Δ*F*) and the normalization (normalized Δ*F*, also called Δ*F*/*k*) from a single participant during a triangular‐shaped contraction (left). Individual discharges and smoothed vector regression of a test (orange) and two control (blue) motor units as a function of time. Brace height measurement (right) is displayed with the discharge rate smoothed vector regression (thin black line), the brace height (blue), the height of the right triangle used to normalize this value (orange), the acceleration slope (green) and the attenuation slope (red). Abbreviations: CTF, cramp threshold frequency; *k*, the estimated maximal hysteresis of the control unit; MVC, maximal voluntary contraction; PIC, persistent inward current; pps, pulses per second; WPHF, wide‐pulse, high‐frequency neuromuscular electrical stimulation.

Then, three measurements to estimate different aspects of PIC contributions to motoneuron discharge were performed. First, WPHF was delivered at the intensity required to elicit 10% MVC torque. The intensity was adjusted with 1 s trains (pulse duration of 1 ms and stimulation frequency of 100 Hz) until consistently reaching a 10% MVC evoked torque response, and two 20 s trains, separated by 2 min rest, were distributed at this intensity. The extra torque and sustained EMG activity were used as indices of PIC strength (Figure 1b). Second, neuromuscular electrical stimulation intensity was set to elicit 20% MVC torque (pulse duration of 1 ms and stimulation frequency of 20 Hz). The participant's Achilles tendon was mechanically vibrated with an amplitude of 1 mm at 100 Hz using a vibrator steadily held by an experimenter at the level of the medial malleolus. After 10 s of vibration, 5 × 2 s stimulation trains, each separated by 2 s (2 s on, 2 s off), were delivered to the triceps surae muscle at the predetermined intensity. The vibration was maintained during the whole 20 s of stimulation and for 3 s after the end of the stimulation (Mesquita et al., 2021). The warm‐up effect was then calculated to estimate the facilitation of PICs (Figure 1c). Third, submaximal triangular voluntary contractions (0%–20%–0% MVC, 10 s up and 10 s down) were performed for HD‐EMG acquisition, before and after the cramp threshold frequency test used to elicit an electrically induced cramp in the gastrocnemius medialis muscle. Specific parameters, such as the delta frequency (Δ*F*) and brace height and associated slopes (e.g., acceleration and post‐acceleration attenuation), were calculated to estimate the contributions of PICs to motoneuron discharge (Figure 1d) and are described below. Only the triangular voluntary contractions were repeated after the cramp‐inducing protocol, because this task enabled evaluation of post‐cramp changes in motor unit discharge behaviour and PIC‐related metrics in controlled voluntary conditions, while minimizing additional electrically evoked activation that could confound post‐cramp adaptations.

### 2.6 Torque measurement

Plantar flexor torque was recorded using a custom‐built footplate equipped with a strain gauge sensor (S2M force transducer, 1000 N, HBM Test & Measurement, Darmstadt, Germany). The Wheatstone bridge sensitivity was 2 mV/V, and the signal was amplified with a gain of 1000 and digitized at 1250 Hz using a Biopac acquisition system (Biopac Systems Inc., USA). The transducer was calibrated with a sensitivity of 0.1 N m/mV, consistent with previous experiments (Popesco et al., 2024). Participants were seated, with the hip, knee and ankle positioned at 90°, and the thigh was stabilized with a padded brace secured above the knee to prevent knee extension and isolate ankle plantar flexion torque (Figure 2a).

**FIGURE 2:**
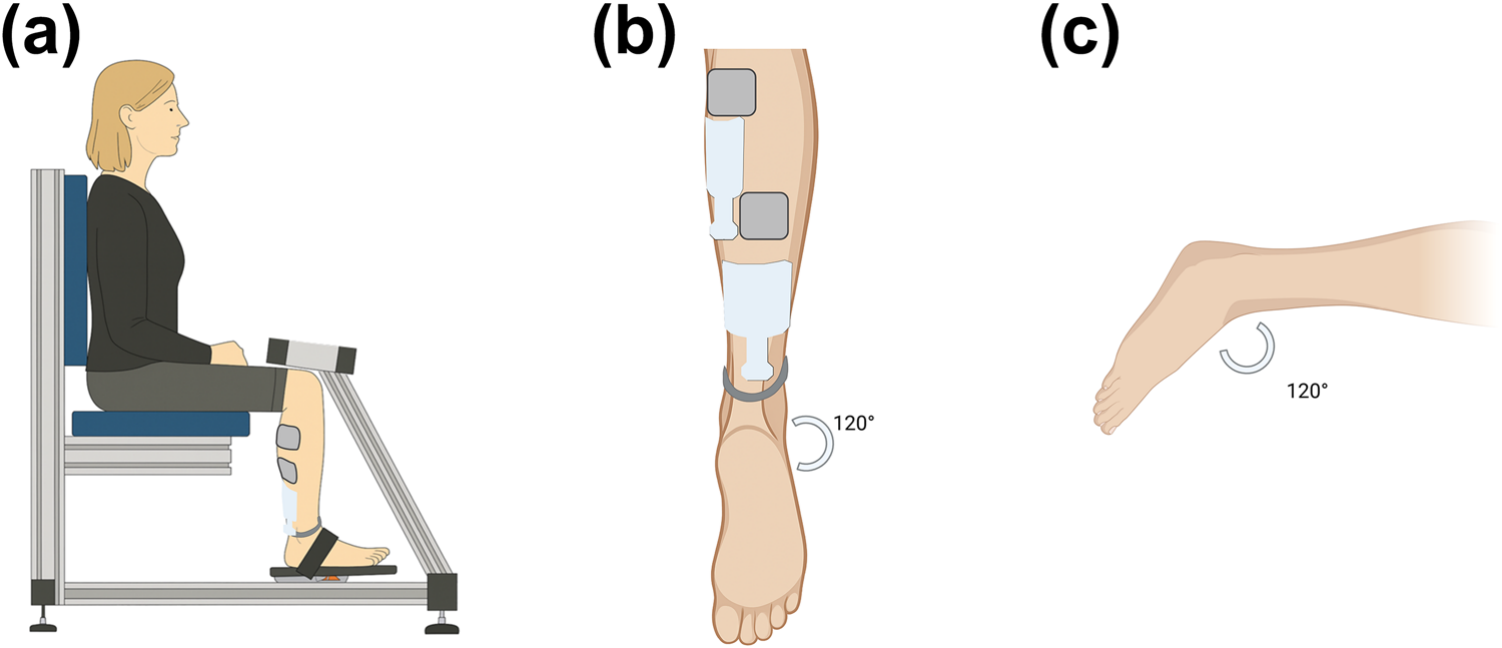
(a) Positioning of the participant in the ergometer. Participants had visual torque feedback in front of them. (b, c) Representation of the leg during the cramp threshold frequency protocol, with a posterior view and the electrodes (b) and a lateral view to show the foot angle (c). Stimulation electrodes (grey squares), HD‐EMG electrodes (white) and the strap used as a reference for HD‐EMG recordings (grey band) are displayed in both panels. Abbreviation: HD‐EMG, high‐density surface EMG.

### 2.7 Neuromuscular electrical stimulation and vibration

Neuromuscular electrical stimulation was delivered transcutaneously by a constant‐current stimulator (DS7AH, Digitimer, Welwyn Garden City, UK). Tibial nerve stimulation (1 ms, 400 V) was used to assess M‐waves, H reflexes and voluntary activation and was applied via surface electrodes (1 cm cathode, Uni‐Patch R series, Covidien) over the popliteal fossa, above the tibial nerve and at the anterior surface of the knee, 2–3 cm proximal to the patella (9 × 5 cm anode, Uni‐Patch R series, Covidien, Dublin, Ireland). WPHF (1 ms, 100 Hz, 400 V) was applied to the triceps surae using large surface electrodes (9 cm × 5 cm, Uni‐Patch R series, Covidien) positioned over the widest part of the gastrocnemii muscles (∼4 cm below the popliteal fossa) and over the soleus below the gastrocnemii muscles. The Achilles tendon was vibrated with an amplitude of 1 mm at 100 Hz by a hand‐held vibrator (VibraMoov, Techno Concept, Manosque, France).

### 2.8 Cramp threshold protocol

Cramp threshold frequency was assessed following the protocol described by Behringer et al. (2014). Participants were positioned lying prone on a table, with the knee fully extended, the foot not restrained but with the ankle supported in slight plantar flexion (∼10°–15°) to minimize passive stretch of the triceps surae. The gastrocnemius medialis muscle of the dominant leg was targeted. Two self‐adhesive surface electrodes (5 cm × 5 cm, Uni‐Patch R series, Covidien) were positioned, with the cathode over the motor point of the gastrocnemius medialis, identified as the point where we could elicit the maximal twitch response at the lowest current, and the anode placed ∼5–10 cm proximally, over the muscle belly or popliteal fossa (Figure 2b). Neuromuscular electrical stimulation was delivered with monophasic rectangular pulses of 0.5 ms duration with the same stimulator used previously (DS7AH, Digitimer). Stimulation trains lasted 5 s, and the current intensity was set to 40 mA and was then held constant throughout the test. The stimulation frequency was increased from 4 Hz in 2 Hz increments, with a 55 s rest period between each train. Participants were instructed to remain fully relaxed throughout and to report immediately any painful or sustained muscle contractions. A cramp was defined as a sudden, involuntary, painful contraction of the muscle that persisted beyond the end of the stimulation train and was confirmed by palpation and participant report. The lowest stimulation frequency that elicited the cramp was recorded as the cramp threshold frequency and was expressed in hertz. If no cramp was induced by 40 Hz, the test was terminated, and cramp threshold frequency was recorded as not obtained. The procedure was discontinued 5 s after cramp onset, with a short stretch of the plantar flexors performed by the experimenter to minimize discomfort and avoid prolonged cramping.

### 2.9 EMG recording

Bipolar surface EMG was recorded using self‐adhesive surface electrodes (Ag/AgCl, Medi‐Trace 100, Tyco Healthcare, Markham, ON, Canada; recording diameter, 1 cm; inter‐electrode distance, 2 cm) placed over soleus and gastrocnemius medialis (2 cm inter‐electrode distance) after skin preparation. Signals were amplified (×1000), bandpass filtered (10–500 Hz), digitized at 5 kHz, and stored for offline analysis (Acqknowledge v.4.2, BIOPAC Systems, Goleta, CA, USA).

HD‐EMG was collected with 64‐channel electrode grids on gastrocnemius medialis (GR08MM1305, OT Bioelettronica) and soleus (GR10MM0808, OT Bioelettronica) placed with adhesive foam and conductive paste after shaving and abrading the skin. The gastrocnemius medialis grid was positioned 1 cm distal to the popliteal crease and 2 cm medial to the junction between gastrocnemius heads (Vinti et al., 2018), and the soleus grid was placed 10 mm below the distal insertion of gastrocnemius medialis (Cohen, 2020). HD‐EMG was acquired with differential amplification (×150), bandpass filtered (10–900 Hz) and digitized (2048 Hz) using a 16‐bit A/D converter (Quattrocento, OT Bioelettronica). HD‐EMG signals were decomposed into individual motor unit spike trains using a convolutive blind source kernel compensation method of separation (Holobar et al., 2014). Decomposition accuracy was refined through manual correction of missed spikes and discharge irregularities (Del Vecchio et al., 2020). Instantaneous discharge rates were estimated from interspike intervals and smoothed using support vector regression (Beauchamp et al., 2023).

### 2.10 Data analysis

#### 2.10.1 Voluntary activation

Voluntary activation was assessed using the twitch interpolation technique, in which a supramaximal electrical stimulus was superimposed on a maximal voluntary contraction and compared with a potentiated twitch delivered at rest. Voluntary activation was expressed as a percentage according to the formula: Voluntary activation (%) = [1 − (superimposed twitch/potentiated twitch)] × 100 (Allen et al., 1995).

#### 2.10.2 Spinal reflex and peripheral excitability

Bipolar EMG outcomes included maximal H‐reflex peak‐to‐peak amplitude (H_max_), maximal M‐wave peak‐to‐peak amplitude (M_max_) and H_max_/M_max_ ratio as indicators of spinal and peripheral excitability. All bipolar EMG variables were extracted from soleus and gastrocnemius medialis muscles.

#### 2.10.3 Responses to WPHF

Extra torque was defined as the absolute difference in torque between the 2^nd^ and 20^th^ seconds (extra torque = 20^th^ second torque − 2^nd^ second torque); Figure 1b; Popesco et al., 2024). Mean torque was calculated as the force produced during stimulation, expressed relative to MVC torque, excluding the first second of stimulation to avoid the initial ascending phase.

Sustained EMG activity (Figure 1b) was computed as the EMG root mean square quantified over 500 ms after the end of the 20 s train of WPHF, starting 200 ms after baseline return, normalized to peak MVC root mean square (Donnelly et al., 2021).

The warm‐up effect was calculated as the absolute difference between the torque in a 500 ms window produced, with vibration only after the five bouts of neuromuscular electrical stimulation (*T*_vib_) and the torque after the first train of stimulation (*T*_1_) (warm‐up effect = *T*_vib_ − *T*_1_; Figure 1c).

#### 2.10.4 HD‐EMG‐derived motor unit discharge metrics

Obtained from HD‐EMG measurements, discharge rate hysteresis (i.e., ∆*F*) of a higher‐threshold motor unit (test unit) relative to a lower‐threshold unit (control unit) was calculated as the average change in control unit discharge rate between test unit recruitment and derecruitment, across all valid pairings (Figure 1d; Hassan et al., 2020). Pairs were included only if they showed strong rate–rate correlation (*R* > 0.7), recruitment time difference > 1 s, and a control unit that modulated its discharge rate by ≥0.5 pps while the test unit was active (Gorassini et al., 2002). The Δ*F* values were then normalized by the estimated maximal hysteresis of the control unit (normalized Δ*F*; Škarabot et al., 2025). Brace height was calculated as the peak deviation from a linear fit of the smoothed discharge rate between recruitment and derecruitment of a motor unit (Figure 1d) and was then normalized to the height of a right‐angled triangle, whose hypotenuse represents a straight line from recruitment (Beauchamp et al., 2023). Acceleration and attenuation slopes were extracted from the ascending phase, with brace height defining the transition point between both slopes. Self‐sustained discharge (SSD) was defined as the persistence of motor unit discharge below the torque associated with its recruitment threshold and was calculated as the relative difference between the durations of the descending and ascending phases, expressed as (descending − ascending)/(descending + ascending) × 100% (Mohammadalinejad et al., 2024). All HD‐EMG variables were computed using an adapted and modified version of the OpenHD‐EMG (Valli, 2023) Python 3 interface (Python Software Foundation, USA).

Task performance during the triangular contractions was evaluated by measuring torque tracking error. Tracking error was defined as the proportion of time during which the produced torque deviated beyond a ±2% MVC bandwidth around the target torque trajectory. The analysis was limited to the active contraction phase (0%–20%–0% MVC), with resting periods excluded.

### 2.11 Statistical analysis

Statistical tests were performed using R (v.4.4.1). Agreement between self‐reported cramp susceptibility (questionnaire) and experimentally induced cramp occurrence (cramp threshold frequency test) was assessed using a 2 × 2 contingency table. Statistical association was tested with Fisher's exact test. Odds ratios with 95% confidence intervals were calculated to estimate the strength of association. Linear mixed models were used to assess the fixed effects of time, group and the time × group interaction on all HD‐EMG variables and torque tracking error. The random effect was specified for individual motor units and for the control motor units in the case of Δ*F*. Data normality was assessed via Shapiro–Wilk tests. Student's *t*‐tests were performed to compare participant characteristics, MVC, voluntary activation and centrally mediated responses to WPHF between groups when normality was verified. Wilcoxon rank tests were used when data were not normally distributed. Sex distribution between groups was compared using Fisher's exact test. Significance was set at *P *< 0.05. Data are reported as the mean ± SD.

## 3 RESULTS

### 3.1 Cramp susceptibility

The cramp threshold frequency protocol evoked a cramp on the gastrocnemius medialis muscle in 10 individuals, of whom 8 were cramp‐prone individuals and 2 were controls based on the questionnaire. On average, the stimulation frequency to evoke a cramp was 22 Hz (range, 12–30 Hz). There was a significant association between self‐reported cramp susceptibility and cramp occurrence during the cramp threshold frequency test (Fisher's exact test, *P* = 0.046). The odds of experiencing a cramp during the test were eight‐fold higher in participants who reported being prone to cramps compared with controls (odds ratio = 8.0).

### 3.2 Participant characteristics

Controls and crampers did not differ for age (25 ± 8 vs. 24 ± 6 years, *P* = 0.828), body mass (68 ± 13 vs. 69 ± 13 kg, *P* = 0.886), height (173 ± 8 vs. 172 ± 8 cm, *P* = 0.786) or sex distribution (control: 12 women and 6 men; cramp: 8 women and 2 men; Fisher's exact test, *P* = 0.669).

Plantar flexor MVC torque was not different between groups (control: 138.2 ± 47.8 N m vs. cramp: 136.2 ± 32.8 N m, *P* = 0.829). Voluntary activation was also not different when comparing controls and crampers (control: 95.2% ± 4.0% vs. cramp: 93.8% ± 6.2%, *P* = 0.303).

### 3.3 Motor unit identification

For the included participants, on average 3.3 ± 2.7 motor units per participant were identified and tracked before and after the cramp protocol in soleus, and 8.1 ± 8.1 motor units per participant were identified in gastrocnemius medialis. On average, 7.0 ± 12.6 motor unit pairs were tracked in soleus and 51.9 ± 80.0 in gastrocnemius medialis. Of these pairs, 2.5 ± 9.1 and 24.6 ± 46.4 Δ*F* values were computed, respectively, after applying the selection criteria.

### 3.4 PIC contributions to motoneuron discharge and excitability

Most PIC‐related variables were comparable between groups, with a significant group × time interaction only in gastrocnemius medialis maximal discharge rate. In soleus, Δ*F* was not significantly different between groups (*P* = 0.735) and remained unchanged over time (time: *P* = 0.431; Figure 3a). In gastrocnemius medialis, Δ*F* was higher in controls compared with crampers and was increased after the elicited cramp, with significant group (*P* < 0.001) and time (*P* = 0.037) effects. No group × time interaction was detected (*P* = 0.857; Figure 3b).

**FIGURE 3:**
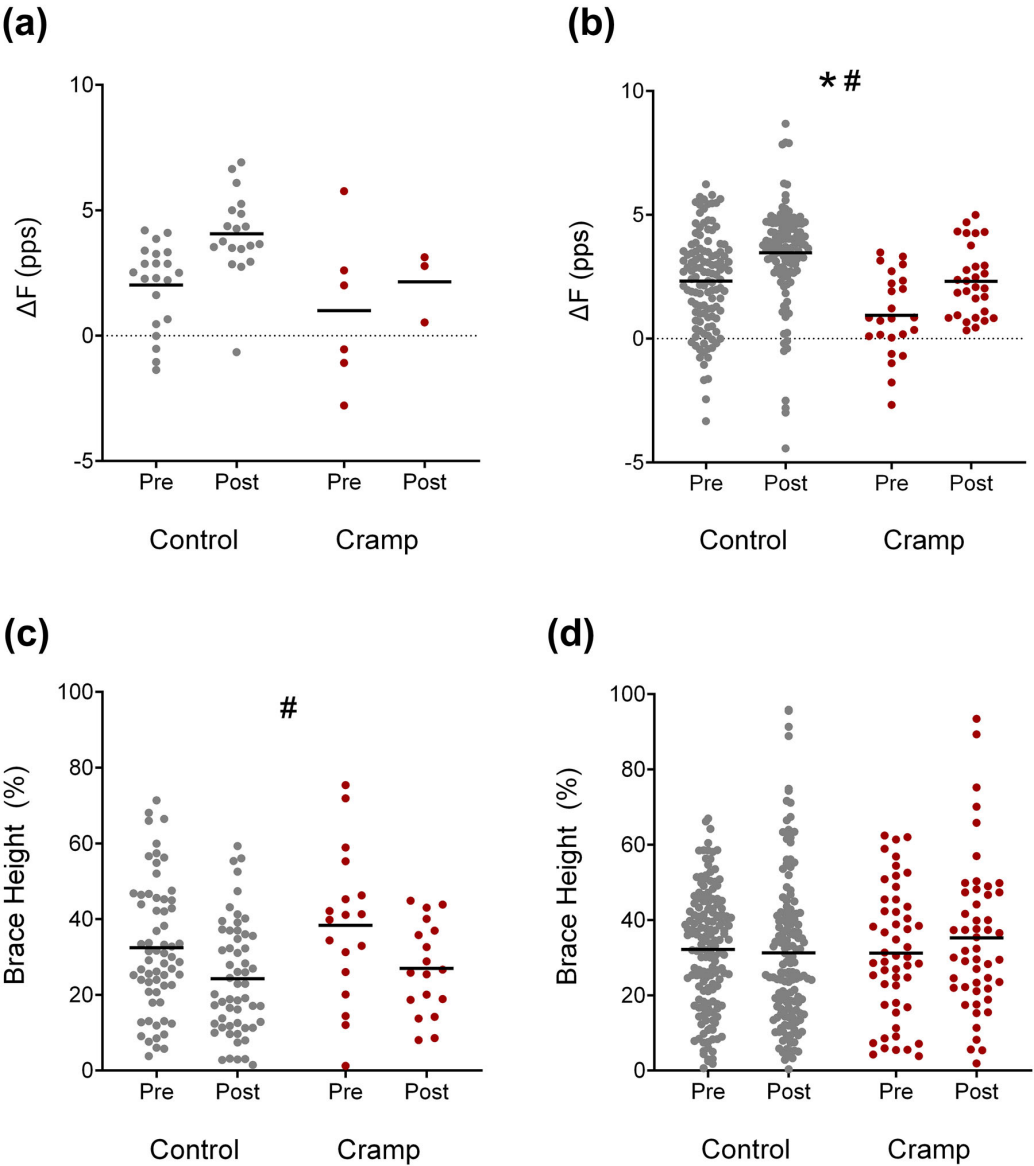
Δ*F* of each motor unit pair for soleus (a) and gastrocnemius medialis (b) and brace height for soleus (c) and gastrocnemius medialis (d) between control and cramp groups. Each point represents one motor unit pair for Δ*F* (a, b) and one motor unit for brace height (c, d). The horizontal black line represents the mean. Sample sizes were as follows: (a) control pre/post: *n* = 21/19, cramp pre/post: *n* = 6/3; (b) control pre/post: *n* = 121/122, cramp pre/post: *n* = 24/29; (c) control pre/post: *n* = 63/56, cramp pre/post: *n* = 18/18; and (d) control pre/post: *n* = 162/151, cramp pre/post: *n* = 51/50. ^*^Main effect of group (*P *< 0.05). ^#^Main effect of time (*P *< 0.05).

In soleus, normalized Δ*F* did not differ between groups (*P* = 0.734) or between time points (*P* = 0.432), with no group × time interaction (*P* = 0.488). In gastrocnemius medialis, normalized Δ*F* increased following the cramp threshold frequency protocol (main effect of time, *P* = 0.006) but did not differ between groups (*P* = 0.712; Table 1).

**TABLE 1:** Maximal discharge rate, normalized Δ*F*, acceleration and attenuation slopes between groups

Parameter		Control		Cramp	
		Pre	Post	Pre	Post
Maximal discharge rate (pps)	Soleus	10.7 ± 3.0	11.6 ± 4.0^#^	10.7 ± 2.1	12.5 ± 4.8^#^
	Gastrocnemius medialis	11.2 ± 2.8	11.7 ± 3.4^†^	10.3 ± 1.9	9.0 ± 2.0
Normalized Δ*F* (%)	Soleus	69.9 ± 36.1	66.3 ± 28.3	72.3 ± 31.6	56.0 ± 32.3
	Gastrocnemius medialis	66.4 ± 31.0	71.1 ± 22.1^#^	61.3 ± 41.9	79.3 ± 26.9^#^
Acceleration slope (pps/%MVC)	Soleus	1.3 ± 1.1	0.9 ± 1.0	1.3 ± 0.6	1.0 ± 0.8
	Gastrocnemius medialis	1.2 ± 0.8	1.0 ± 1.0	1.0 ± 0.5	1.2 ± 1.4
Attenuation slope (pps/%MVC)	Soleus	0.2 ± 0.2	0.2 ± 0.2	0.2 ± 0.3	0.2 ± 0.3
	Gastrocnemius medialis	0.2 ± 0.6	0.1 ± 0.4	0.2 ± 0.2	0.0 ± 0.5

Abbreviations: ΔF, change in discharge rate of the lower‐threshold control motor unit between recruitment and derecruitment of the higher‐threshold test motor unit; %MVC, percentage of maximal voluntary contraction torque.

^#^ Main effect of time (*P* < 0.05).

^†^ Group × time interaction (*P* < 0.05).

In soleus, brace height was not different between groups (*P* = 0.084) but decreased over time (*P* < 0.001; Figure 3c). In gastrocnemius medialis, brace height was not different between groups or time points (*P* = 0.656 and *P* = 0.162; Figure 3d).

In both muscles, SSD was significantly different between time points, with a higher value at post (soleus, *P* = 0.004 and gastrocnemius medialis, *P* = 0.003; Figure 4), whereas it was not different between groups (soleus, *P* = 0.145 and gastrocnemius medialis, *P* = 0.294).

**FIGURE 4:**
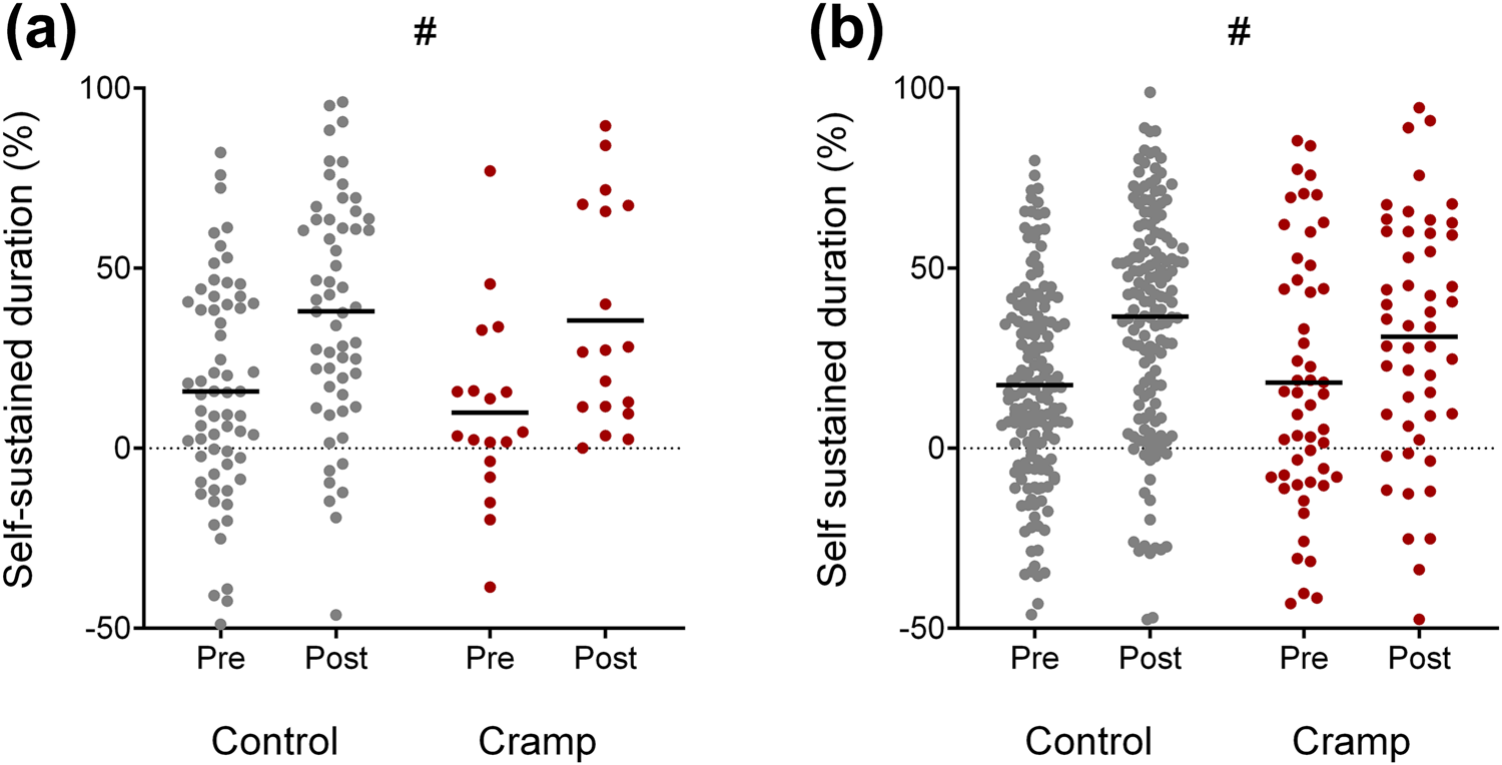
Self sustained duration of each motor unit pair for soleus (a) and gastrocnemius medialis (b) between control and cramp groups. Each point represents one motor unit. The horizontal black line represents the mean. Sample sizes were as follows: (a) control pre/post: *n* = 63/56, cramp pre/post: *n* = 18/18; and (b) control pre/post: *n* = 162/151, cramp pre/post: *n* = 51/50. ^#^Main effect of time (*P *< 0.05).

In soleus, maximal discharge rate during the voluntary triangular contraction did not differ between groups (*P* = 0.981) but increased from pre to post across both groups (main effect of time, *P* = 0.004). In gastrocnemius medialis, maximal discharge rate decreased after the cramp threshold frequency protocol in crampers (*P* = 0.015) but remained unchanged in controls (*P* = 0.590). There was no group difference at baseline (*P* = 0.516), whereas maximal discharge rate was higher in controls than in cramp‐prone individuals at post (*P* < 0.001).

In both soleus and gastrocnemius medialis, acceleration and attenuation slopes were not different between groups (respectively, soleus, *P* = 0.914 and *P* = 0.402 and gastrocnemius medialis, *P* = 0.391 and *P* = 0.927) or between time points (respectively, soleus, *P* = 0.450 and *P* = 0.428 and gastrocnemius medialis, *P* = 0.163 and *P* = 0.070; Table 1).

In both soleus and gastrocnemius medialis, there were no baseline differences for H_max_ (soleus: control, 5.19 ± 2.32 mV and cramp, 4.94 ± 2.45 mV, *P* = 0.800; and gastrocnemius medialis: control, 2.27 ± 1.40 mV and cramp, 2.19 ± 1.32 mV, *P* = 0.981), M_max_ (soleus: control, 8.90 ± 2.88 mV and cramp, 7.01 ± 2.31 mV, *P* = 0.074; and gastrocnemius medialis: control, 5.54 ± 1.83 mV and cramp, 5.88 ± 1.87 mV, *P* = 0.655), H_max_/M_max_ (soleus: control, 60.16% ± 22.41% and cramp, 78.99% ± 43.08%, *P* = 0.225; and gastrocnemius medialis: control, 46.46% ± 30.88% and cramp, 41.69% ± 25.65%, *P* = 0.981) between the controls and the crampers.

Torque tracking error did not differ between groups or time points, and no group × time interaction was observed (all *P* > 0.15), with values of 4.7% ± 10.3% and 1.8% ± 2.6% in controls, and 1.7 ± 1.8% and 0.7 ± 1.0% in crampers, before and after the cramp‐inducing protocol, respectively.

### 3.5 WPHF‐evoked responses between groups

WPHF mean torque (controls, 14.6% ± 6.9% MVC torque vs. crampers, 24.5% ± 21.4% MVC torque, *P* = 0.172) and extra torque (controls, −22.8% ± 55.0% MVC torque vs. crampers, 9.1% ± 108.2% MVC torque, *P* = 0.683) were not different between groups (Figures 5a and 6a,b).

**FIGURE 5:**
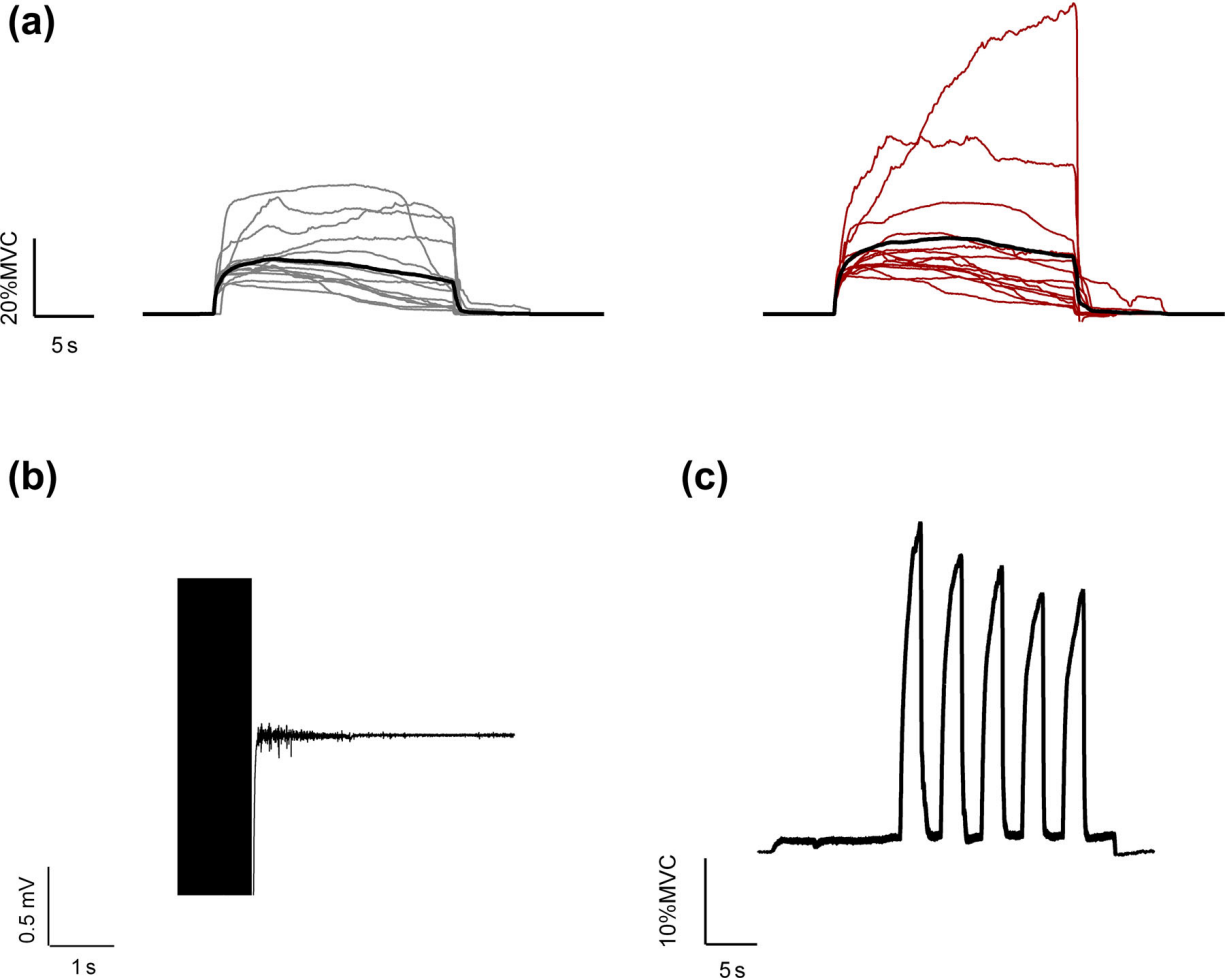
(a) Original recordings of torque in response to WPHF for all controls (grey) and crampers (red). The black lines represent the mean for each group. (b) Recordings of soleus sustained EMG activity from one representative participant. (c) Original torque traces of one representative participant during the vibration combined with neuromuscular electrical stimulation. Abbreviations: MVC, maximal voluntary contraction; WPHF, wide‐pulse, high‐frequency neuromuscular electrical stimulation.

**FIGURE 6:**
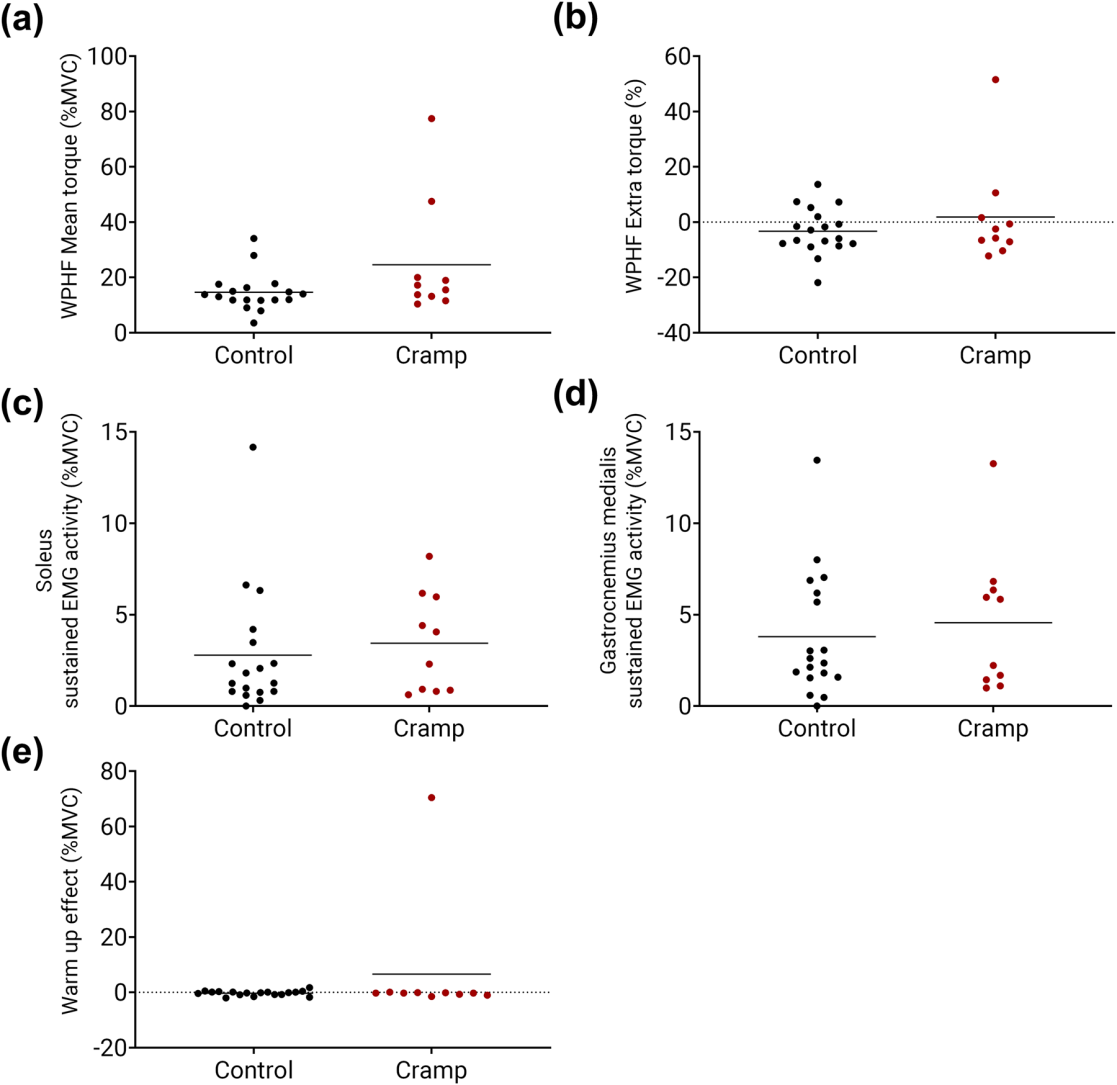
WPHF mean torque (a), extra torque (b) and sustained EMG activity for soleus (c) and gastrocnemius medialis (d) and warm‐up effect (e) between control and cramp groups. Each point represents one participant. The horizontal black line represents the mean. Sample sizes were *n* = 18 controls and *n* = 10 crampers for all panels. Abbreviations: MVC, maximal voluntary contraction; WPHF, wide‐pulse, high‐frequency neuromuscular electrical stimulation.

Sustained EMG activity was not different between groups for both soleus (2.8% ± 3.4% vs. 3.4% ± 2.7% maximal EMG activity, *P* = 0.401) and gastrocnemius medialis (3.8% ± 3.4% vs. 4.6% ± 3.9% maximal EMG activity, *P* = 0.905; Figures 5b and 6c,d). The warm‐up effect measured in response to neuromuscular electrical stimulation superimposed on tendon vibration did not differ significantly between groups (controls, −0.3% ± 0.9% MVC vs. cramp, 6.6% ± 22.4% MVC, *P* = 0.615; Figures 5c and 6e).

## 4 DISCUSSION

In the present study, we investigated whether susceptibility to cramp is associated with altered motoneuron excitability at baseline and whether an electrically induced muscle cramp modulates contributions of PICs and motor unit discharge behaviour differently between crampers and non‐crampers. We hypothesised that crampers would exhibit greater baseline contributions of PICs to motoneuron discharge, namely higher WPHF‐evoked torque, greater sustained EMG responses to WPHF and enhanced warm‐up effect, and distinct post‐cramp changes in motor unit discharge behaviour compared with controls. Overall, our findings support these hypotheses only in part. At baseline, most HD‐EMG‐derived estimates of PIC contributions to motoneuron discharge and H‐reflex measures did not differ between groups, although Δ*F* in gastrocnemius medialis was lower in crampers than in controls. Following the cramp‐inducing protocol, Δ*F* increased in gastrocnemius medialis and SSD increased in both muscles, whereas brace height remained unchanged in gastrocnemius medialis but decreased in soleus in both groups. In addition, maximal discharge rate in gastrocnemius medialis decreased in crampers but not in controls. WPHF‐evoked torque, sustained EMG activity and the warm‐up effect did not differ between groups. Taken together, these results suggest that the cramp‐inducing protocol induced subtle, muscle‐specific changes in motor unit discharge behaviour across participants, while only limited aspects of discharge modulation differed between crampers and non‐crampers.

### 4.1 Susceptibility to muscle cramp

Although no cramp was elicited during the cramp threshold frequency test in some individuals who self‐reported being prone to cramps, electrically induced cramps were significantly associated with self‐reported cramp susceptibility (Fisher's exact test, *P* = 0.046). The odds of cramping during the test were markedly higher in participants who reported being prone to cramps, supporting the relevance of the cramp threshold frequency protocol as an indicator of cramp susceptibility, even if not all self‐reported crampers exhibited cramps during testing, as also observed by Harmsen et al. (2021). This relationship is consistent with earlier findings that lower cramp threshold frequencies are associated with greater habitual cramp frequency (Miller & Knight, 2009; Minetto et al., 2011). Although cramp threshold frequency reflects the minimum stimulation frequency required to elicit a sustained involuntary contraction, it might also capture individual differences in spinal excitability or muscle fibre membrane properties (Behringer et al., 2018; Bertolasi et al., 1993). However, the absence of any difference in the H_max_/M_max_ ratio between groups reinforces previous evidence indicating that spinal excitability at rest does not differ between controls and crampers (Harmsen et al., 2021). The fact that some self‐reported crampers did not cramp during the cramp test highlights that susceptibility to muscle cramp is multifactorial and might depend on the interaction between the acute physiological state and prior cramp history (Schwellnus, 2009).

### 4.2 MVC and voluntary activation at baseline

MVC torque and voluntary activation were assessed at baseline to determine whether global neuromuscular output differed between groups. MVC torque did not differ between controls and crampers, and voluntary activation was also comparable between groups, indicating similar capacity to drive the plantar flexor muscles voluntarily. No previous study has directly examined differences in MVC torque and voluntary activation between individuals prone to muscle cramps and controls; however, it has been suggested that mechanisms underlying muscle cramps should not substantially influence these global measures of maximal force production (Maughan & Shirreffs, 2019; Roeleveld et al., 2000). These findings suggest that cramp susceptibility is unlikely to be explained by differences in the ability to recruit the motoneuron pool voluntarily. The absence of group differences in these global measures reinforces the notion that cramp susceptibility might arise from more subtle, muscle‐specific differences in motor unit discharge behaviour rather than from deficits in overall motor drive or maximal torque generation.

### 4.3 Differences between groups at baseline

At baseline, most motor unit discharge metrics were similar between controls and crampers in both soleus and gastrocnemius medialis. Brace height, normalized Δ*F* and acceleration and attenuation slopes did not differ between groups in both soleus and gastrocnemius medialis. This suggests that global PIC‐related gain and the overall trajectory of discharge modulation were not altered in crampers compared with controls (Beauchamp et al., 2023). However, Δ*F* in gastrocnemius medialis was significantly lower in crampers than in controls, whereas Δ*F* in soleus did not differ between groups. Because Δ*F* is widely interpreted as an indirect estimate of PIC‐mediated motor unit recruitment–derecruiment hysteresis, reduced gastrocnemius medialis Δ*F* in crampers might indicate a somewhat lower contribution of PICs to discharge modulation in this muscle (Mesquita et al., 2021; Orssatto et al., 2021; Popesco, Bet da Rosa Orssatto et al., 2024).

The fact that these between‐group differences emerged only in gastrocnemius medialis, and not in soleus, is consistent with previous reports of muscle‐specific variations in motoneuron behaviour and PIC expression (Heckman & Enoka, 2012; Revill & Fuglevand, 2017). Because gastrocnemius medialis is more frequently implicated in cramps than soleus in both experimental and spontaneous contexts (Behringer et al., 2018; Harmsen et al., 2021; Miller & Knight, 2009; Minetto et al., 2011), even relatively subtle alterations in its discharge dynamics might contribute to a greater propensity for cramp initiation under high synaptic drive. At the same time, spinal reflex excitability at rest, as reflected by the H_max_/M_max_ ratio, did not differ between groups, consistent with earlier studies showing comparable H‐reflex behaviour between controls and crampers (Harmsen et al., 2021). Taken together, these findings suggest that cramp susceptibility is not associated with a uniform increase in baseline spinal reflex excitability or PIC‐related gain, but rather with more nuanced, muscle‐specific differences in motor unit discharge modulation.

Contrary to our initial hypothesis, WPHF‐evoked mean torque, extra torque, sustained EMG activity and the warm‐up effect did not differ significantly between groups. This indicates that crampers do not exhibit systematic reflexive torque amplification or sustained EMG responses in the WPHF and vibration conditions used here. Given the large interindividual variability typically observed in WPHF responses (Collins, 2007; Dean et al., 2007; Popesco, Gardet et al., 2024; Wegrzyk et al., 2015), it is possible that subtle group differences, if present, were reduced by the variability in afferent feedback, inhibitory interneuron activity, descending neuromodulatory tone or peripheral muscle properties. Nevertheless, the lack of group differences in these evoked responses, combined with similar H‐reflex amplitudes, argues against a simple model in which cramp susceptibility arises from a global upregulation of reflex excitability or PIC amplification at rest. Instead, the present data support muscle‐specific adjustments of motor unit discharge dynamics, such as those observed in gastrocnemius medialis Δ*F* and acceleration slope.

### 4.4 Effect of muscle cramp induction on motoneuron behaviour

The cramp‐inducing protocol produced several acute changes in motor unit discharge behaviour, but most of these effects were not specific to crampers. In gastrocnemius medialis, both Δ*F* and normalized Δ*F* increased across time, indicating a modest but consistent shift in the shape of motor unit discharge modulation and discharge rate hysteresis of motor units after repetitive stimulation and cramp induction. These changes are compatible with a greater contribution of PICs to overall discharge modulation and a subtle reorganization of the ascending discharge rate trajectory following strong afferent and synaptic activation (Beauchamp et al., 2023; Collins, 2007; Škarabot et al., 2025). However, the absence of a significant group × time interaction suggests that these adaptations occurred in both controls and crampers and might therefore reflect general neuromodulatory effects of the cramp threshold frequency protocol rather than mechanisms specific to cramp susceptibility.

In soleus, Δ*F* and normalized Δ*F* remained unchanged across time, whereas brace height decreased following the cramp threshold frequency protocol and maximal discharge rate increased modestly, with no evidence of a group‐specific effect. Acceleration and attenuation slopes also remained stable. Together, these findings indicate that although the overall magnitude of PIC‐related hysteresis was preserved, the geometric features of the discharge rate trajectory were subtly altered after the protocol, reflecting changes in the balance of excitatory and inhibitory commands (Beauchamp et al., 2023; Škarabot et al., 2025). Such dissociations between PIC magnitude and discharge trajectory geometry have been reported following strong synaptic or neuromodulatory activation and are thought to reflect adjustments in how motoneurons regulate discharge rate modulation rather than changes in intrinsic PIC gain (Heckman et al., 2009; Revill & Fuglevand, 2017). Importantly, the absence of group differences in these soleus adaptations suggests that the cramp‐inducing protocol elicited a general reorganization of discharge patterns in the synergist muscle, rather than a response specific to cramp susceptibility (Harmsen et al., 2021; Minetto et al., 2011).

In contrast, maximal discharge rate in gastrocnemius medialis exhibited a clear group‐dependent response. In crampers, maximal discharge rate decreased following the cramp threshold frequency protocol, whereas no change was observed in controls. This suggests that cramp‐prone motoneurons might respond differently to strong involuntary activation and afferent input, potentially reflecting an altered regulation of discharge behaviour following the cramp‐inducing protocol. Such a reduction in maximal firing capacity might reflect transient changes in motoneuronal responsiveness, synaptic integration or inhibitory mechanisms following repetitive activation. Although the present study cannot determine the precise mechanisms, these findings suggest that cramp susceptibility might be associated with a different adaptation of motor unit discharge behaviour following intense activation.

In both soleus and gastrocnemius medialis, SSD increased significantly from pre to post, independently of group. Because SSD reflects the capacity of motoneurons to maintain discharge as synaptic drive declines (Afsharipour et al., 2020), this systematic increase suggests that brief, high‐intensity involuntary muscle activation enhanced discharge persistence across muscles. Notably, this effect occurred despite divergent changes observed in other PIC‐related indices. The lack of a group × time interaction indicates that this effect was not specific to crampers but rather reflects a general adaptation of motoneuron behaviour to the experimental protocol, consistent with the idea that strong afferent and neuromodulatory inputs can enhance PIC activation and self‐sustained discharge even in the absence of overt group differences in baseline excitability. Importantly, torque tracking error during voluntary contractions was not altered after the cramp‐inducing protocol, suggesting that the observed modulation of PIC‐related discharge behaviour did not translate into a measurable impairment of torque control or task‐level gain regulation.

### 4.5 Mechanistic considerations

Taken together, our findings do not support the notion that cramp susceptibility is driven by a generalized increase in spinal excitability or PIC‐related gain. At baseline, most PIC‐related motor unit metrics were comparable between groups, and differences were limited to a lower Δ*F* in crampers. These subtle, muscle‐specific differences support a model in which cramp susceptibility reflects localized changes in how motor unit discharge is modulated within certain motor pools (Heckman & Enoka, 2012), rather than a global alteration of intrinsic motoneuron properties as observed in some pathological conditions (ElBasiouny et al., 2010; Leroy et al., 2014).

The cramp‐inducing protocol elicited small but consistent increases in PIC‐related indices in gastrocnemius medialis (Δ*F* and normalized Δ*F*) and in SSD for both muscles, along with a reduction in gastrocnemius medialis attenuation slope, consistent with a generalized facilitation of self‐sustained discharge following intense activation (Orssatto et al., 2021, 2023). These adaptations were similar in controls and crampers, suggesting that both groups responded to the protocol with a comparable enhancement of PIC‐related behaviour. The main group‐dependent effect was a selective increase in maximal discharge rate of gastrocnemius medialis in controls but not in crampers. This suggests a reduced capacity of cramper motoneurons to increase their discharge output further following intense activation. Such a constraint might reflect stronger recurrent inhibition, altered sodium or calcium conductances, or differences in descending neuromodulatory input, all of which could limit firing rate expansion despite preserved PIC‐related facilitation (Heckman et al., 2009; Lee & Heckman, 1998; Revill & Fuglevand, 2017). Such a reduction in flexibility might predispose these motoneurons to remain in discharge states that are more susceptible to cramp generation during conditions of high synaptic drive, but future studies are needed to test this directly.

### 4.6 Limitations

Several limitations should be considered when interpreting these findings. First, although 28 participants were recruited, the number of identified motor units per participant, particularly in soleus, was relatively low. This is likely to have reduced the precision of some Δ*F* and geometric measures, which might have limited our ability to detect small between‐group differences, especially when values were variable. Second, the cramp threshold frequency test used a fixed stimulation intensity across individuals, which might bias cramp induction: in some participants with low excitability or higher thresholds, the protocol might have underestimated cramp susceptibility or failed to elicit a cramp, whereas in others it might have induced unusually strong contractions. Third, electrically induced cramps in a controlled laboratory setting might not fully replicate the physiological conditions underlying spontaneous exercise‐associated or nocturnal cramps, which often involve fatigue, metabolic perturbation or environmental stressors (Schwellnus, 2009). Finally, although we used multiple motor‐unit‐level and evoked measures of motoneuron excitability, these remain indirect estimates of PIC behaviour and cannot capture all aspects of spinal and supraspinal control relevant to cramp susceptibility.

## 5 CONCLUSION

In summary, crampers did not display a generalized increase in spinal reflex excitability or PIC‐related gain compared with controls. Most PIC‐related motor unit metrics and H‐reflex measures were similar between groups, although Δ*F* and acceleration slope in gastrocnemius medialis differed modestly, suggesting muscle‐specific differences in discharge modulation. The cramp‐inducing protocol elicited a general facilitation of PIC‐related behaviour, reflected by increases in Δ*F* and normalized Δ*F* in gastrocnemius medialis and in SSD in both muscles, together with a decrease in gastrocnemius medialis attenuation slope. These adaptations were largely comparable between groups, with the notable exception of gastrocnemius medialis maximal discharge rate, which increased only in controls. WPHF‐evoked torque, extra torque, sustained EMG activity and the warm‐up effect did not differ between groups and were not strongly modulated by the protocol.

Collectively, these findings indicate that cramp susceptibility is not primarily associated with a global elevation of motoneuronal excitability, but might be linked to more subtle, muscle‐specific differences in the dynamics and adaptability of motor unit discharge, particularly in the gastrocnemius. Future studies should examine whether interventions targeting motor unit discharge behaviour and PIC‐related properties can modify cramp occurrence in different physiological conditions. In particular, HD‐EMG recordings obtained during voluntarily induced cramp episodes would allow characterization of discharge rate variability, maximal discharge rates achieved during cramps, and the subset of motor units that remain active, thereby providing direct insight into the motor‐unit‐level mechanisms underlying cramp generation.

## AUTHOR CONTRIBUTIONS

Chris Donnelly, Bengt Kayser and Nicolas Place conceived and designed the study. Timothée Popesco, Romain Dos Santos and Ilan Cassoli collected the data. Timothée Popesco performed the data analysis. Timothée Popesco, Chris Donnelly, Gabriel, S. Trajano, Gregory, E. P. Pearcey, Nicola, A. Maffiuletti, Bengt Kayser and Nicolas Place interpreted the data. Timothée Popesco and Nicolas Place drafted the manuscript. Timothée Popesco, Romain Dos Santos, Ilan Cassoli, Gabriel, S. Trajano, Gregory, E. P. Pearcey, Nicola, A. Maffiuletti, Chris Donnelly, Bengt Kayser and Nicolas Place reviewed and edited the manuscript. All authors critically revised the manuscript for important intellectual content and approved the final version. All authors agree to be accountable for all aspects of the work in ensuring that questions related to the accuracy or integrity of any part of the work are appropriately investigated and resolved. All persons designated as authors qualify for authorship, and all those who qualify for authorship are listed.

## CONFLICT OF INTEREST

None declared.

## GENERATIVE AI STATEMENT

The authors confirm that no artificial intelligence tools, including large language models (LLMs), were used in the drafting of this manuscript. Artificial intelligence was used solely for language editing and correction during the revision process. All content was conceived, written and approved by the authors.

## Data Availability

The data supporting the findings of this study are openly available on Zenodo at https://doi.org/10.5281/zenodo.20629907.
